# Palmitoleic acid reduces high fat diet-induced liver inflammation by promoting PPAR-γ-independent M2a polarization of myeloid cells

**DOI:** 10.1016/j.bbalip.2020.158776

**Published:** 2020-10

**Authors:** Camila O. Souza, Alexandre A.S. Teixeira, Luana Amorim Biondo, Loreana Sanches Silveira, Cristiane N. de Souza Breda, Tarcio T. Braga, Niels O.S. Camara, Thiago Belchior, William T. Festuccia, Tiego A. Diniz, Glaucio Monteiro Ferreira, Mario Hiroyuki Hirata, Adriano B. Chaves-Filho, Marcos Y. Yoshinaga, Sayuri Miyamoto, Philip C. Calder, Jaswinder K. Sethi, José C. Rosa Neto

**Affiliations:** a Department of Cell and Developmental Biology, Institute of Biomedical Sciences, University of São Paulo, São Paulo, SP, Brazil; b Department of Immunology, Institute of Biomedical Sciences, University of São Paulo, São Paulo, SP, Brazil; c Department of Physiology and Biophysics, Institute of Biomedical Sciences, University of São Paulo, São Paulo, SP, Brazil; d Laboratory of Molecular Biology applied to Diagnosis (LBMAD), Department of Pharmacy, Faculty of Pharmaceutical Sciences, University of São Paulo, São Paulo, SP, Brazil; e Department of Biochemistry, Institute of Chemistry, University of São Paulo, SP, Brazil; f Human Development and Health, Faculty of Medicine, University of Southampton, Southampton, UK; g National Institute for Health Research Southampton Biomedical Research Centre, University of Southampton and University Hospital Southampton National Health Service (NHS) Foundation Trust, Southampton, UK; h Institute for Life Sciences, University of Southampton, Southampton, UK

**Keywords:** Palmitoleic acid, Obesity, Non-alcoholic fatty liver disease, Inflammation, Hepatic macrophages

## Abstract

Palmitoleic acid (POA, 16:1n-7) is a lipokine that has potential nutraceutical use to treat non-alcoholic fatty liver disease. We tested the effects of POA supplementation (daily oral gavage, 300 mg/Kg, 15 days) on murine liver inflammation induced by a high fat diet (HFD, 59% fat, 12 weeks). In HFD-fed mice, POA supplementation reduced serum insulin and improved insulin tolerance compared with oleic acid (OA, 300 mg/Kg). The livers of POA-treated mice exhibited less steatosis and inflammation than those of OA-treated mice with lower inflammatory cytokine levels and reduced toll-like receptor 4 protein content. The anti-inflammatory effects of POA in the liver were accompanied by a reduction in liver macrophages (LM, CD11c^+^; F4/80^+^; CD86^+^), an effect that could be triggered by peroxisome proliferator activated receptor (PPAR)-γ, a lipogenic transcription factor upregulated in livers of POA-treated mice. We also used HFD-fed mice with selective deletion of PPAR-γ in myeloid cells (PPAR-γ KO^LyzCre+^) to test whether the beneficial anti-inflammatory effects of POA are dependent on macrophages PPAR-γ. POA-mediated improvement of insulin tolerance was tightly dependent on myeloid PPAR-γ, while POA anti-inflammatory actions including the reduction in liver inflammatory cytokines were preserved in mice bearing myeloid cells deficient in PPAR-γ. This overlapped with increased CD206^+^ (M2a) cells and downregulation of CD86^+^ and CD11c^+^ liver macrophages. Moreover, POA supplementation increased hepatic AMPK activity and decreased expression of the fatty acid binding scavenger receptor, CD36. We conclude that POA controls liver inflammation triggered by fat accumulation through induction of M2a macrophages independently of myeloid cell PPAR-γ.

## Highlights

• Palmitoleic acid (POA) supplementation reduced serum insulin and improved insulin tolerance;
• Livers of POA-treated mice exhibited less steatosis and inflammation;
• POA lowered the liver M1 macrophages population and the expression of inflammation-related immune-cell markers;
• POA increased PPAR-γ, a transcription factor that regulates anti-inflammatory effects in macrophages;
• However, POA reduced liver inflammation even in mice that lack PPAR-γ expression in myeloid cells;
• POA controls liver inflammation through induction of M2a macrophages independently of PPAR-γ in myeloid cells.

## Graphical abstract

**Figure:**
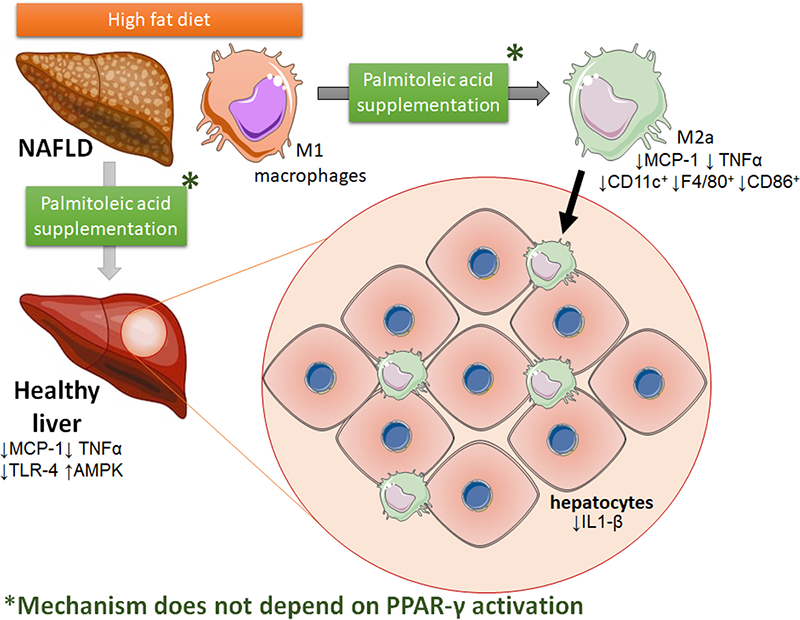

## 1 Introduction

Liver diseases are a major public health problem caused in part by modern lifestyle choices, such as consumption of alcohol and highly caloric diets. With the marked increase in overweight and obese individuals, the incidence of liver diseases has also dramatically increased [1]. Obesity-linked liver disease is generally characterized by ectopic storage of lipids in hepatocyte parenchyma [2,3]. This condition denominated as non-alcoholic fatty liver disease (NAFLD) has an estimated prevalence of *approximately* 40% of the adult population in western countries [4,5]. If not properly treated, NAFLD can advance to non-alcoholic steatohepatitis (NASH, defined by the co-occurrence of fatty liver with inflammation), followed by cirrhosis (advanced fibrosis linked to liver failure) and/or to hepatocellular carcinoma [6].

The liver contains antigen presenting cells, such as macrophages and dendritic cells [7]. The main subtype of liver macrophage is a resident macrophage called the Kupffer cell (Kc). Kupffer cells originate from the fetal liver and are responsible for clearing gut-derived pathogens, and regulate iron, bilirubin and cholesterol metabolism [7]. When activated by aseptic chronic inflammatory signals and/or liver injury, these Kcs also increase the recruitment of granulocyte monocyte progenitor-derived macrophages and dendritic cells from bone marrow to the liver [8,9]. During NAFLD progression these myeloid-derived macrophages are essential to the establishment of fibrosis together with more pro-inflammatory cytokine production [8]. Hepatic infiltration of innate immune cells (neutrophils and macrophages), exacerbates liver metabolic dysfunction, *i*.*e*. insulin resistance, enhances lipogenesis and gluconeogenesis [10].

Few treatments for NAFLD are available, and life-style modification is the main recommendation. However, current evidence suggests that the type of fatty acids ingested in the diet is an important determinant of NAFLD metabolic and inflammatory disturbances [11]. Indeed, a higher dietary content of saturated fatty acids, exhibited by western-style diets, promotes hepatic fat accumulation, contributes to lipotoxicity and increased hepatic inflammation [12]. Conversely, in 2008, Cao and colleagues identified and proposed palmitoleic acid (POA; 16:1n-7) as an adipose-derived ‘lipid hormone’ or lipokine that can strongly stimulate muscle insulin action and suppress hepatic steatosis. Moreover, adipose-derived POA levels remain strongly elevated in a models that remains insulin sensitive despite developing diet-induced obesity [13].

POA is a non-essential fatty acid, produced by desaturation of the carbon 9 of palmitate by stearoyl Co-A desaturase-1 [14]. POA can be found in circulating lipoproteins and cell membranes [15]. It has been extensively studied and shown to mitigate several immune-metabolic alterations caused by lipotoxicity [12,13,[15], [16], [17], [18], [19], [20]]. The beneficial effects of POA supplementation in obesity include: improvement of glucose metabolism and insulin sensitivity in adipose tissue, skeletal muscle, pancreas and liver; restoration of insulin-mediated lipolysis in adipose tissue; and reduction of inflammation in liver, endothelial cells, and macrophages [12,13,16,[20], [21], [22]]. Specifically in liver, POA supplementation reduces inflammation [12,23], improves cholesterol metabolism [24], and reverses insulin resistance through improvement in hepatic glucose metabolism [21]. Furthermore, beneficial effects of POA on liver lipid storage were demonstrated in a genetic model of type 2 diabetes [17].

At the molecular level, POA can influence a number of signaling pathways that target metabolically relevant transcription factors expressed in the liver. We have previously shown that POA supplementation improves liver glucose homeostasis and fatty acid oxidation by activation of peroxisome proliferator activated receptor (PPAR)-α and fibroblast growth factor 21 [21]. However, the anti-inflammatory effects of POA in rodent models of NAFLD/NASH do not seem to require PPAR-α [12]. Another transcription factor increased in the liver with lipid overload and steatosis is PPAR-γ and this may have cell-specific effects. In hepatocytes, PPAR-γ has steatogenic effects, being a major regulator of lipogenesis and an inhibitor of fatty acid oxidation; effects partially mediated by acetyl CoA carboxylase-1 upregulation [25]. In macrophages, PPAR-γ is also a major regulator of pro-resolution and anti-inflammatory responses [26,27]. Here it inhibits nuclear factor kappa B (NFκB) activity, and suppresses the generation of pro-inflammatory cytokines and chemokines [28,29]. Moreover, induced PPAR-γ expression in bone marrow-derived macrophages, promotes macrophage polarization to the M2 phenotype [30]. Importantly, the actions of POA are similar to these anti-inflammatory effects *in vivo* (in whole liver) [12,23], and in cultured macrophages [18]. Despite these beneficial properties, it is not clear if POA supplementation promotes anti-inflammatory effects on hepatic immune-cells activated by diet-induced obesity, and whether PPAR-γ in myeloid cells is required. Therefore, this study aimed to elucidate the role of, and potential requirement for PPAR-γ in myeloid cells as a mediator of the beneficial effects of POA supplementation in diet-induced obesity and NALFD.

## 2 Materials and methods

### 2.1 Animal procedures and diets

Male C57BL/6J wild type (WT), PPAR-γ Flox mice (004584) and Lysozyme MCre (LysCre) mice (004781) were obtained from the Jackson Laboratory and maintained on a 12:12-h light-dark cycle (lights on at 06:00). Beginning at 10 weeks of age, the mice were fed a standard diet (SD, 9% calories from fat) or an modified high-fat diet (HFD, 59% calories from hydrogenated vegetable fat) (Table S1, Table S2) [31]. Mice were weighed weekly for 12 weeks, and in the last two weeks, the HFD-fed mice were treated daily with either oleic acid (OA; 300 mg/kg of body weight (bw)) or POA (300 mg/kg of bw) by oral gavage. The doses and treatment were based on previous studies [16,17]. After the dietary and treatment periods, blood and tissue samples were collected and stored for further analysis. The experimental protocols were approved by the Ethics Committee for Animal Experimentation from the University of São Paulo (049.05.03).

### 2.2 Glucose (GTT), insulin (ITT) and pyruvate (PTT) tolerance tests

After 12 days of treatment with OA or POA, mice previously fasted for 6 h (GTT and ITT) or 16 h (PTT) received i.p. injection of glucose (1.5 g/kg of bw) for GTT, insulin (0.5 U/Kg of bw) for ITT, or sodium pyruvate (2 g/kg of bw) for PTT. Blood samples (5 μl) were collected from the tail vein at specified time points and the levels of glucose were measured by Accu-Chek® performa glucometer (ROCHE®, São Paulo, SP, Brazil). Differences in glycemia before and during i.p. administrations were used to calculate the areas under the curve (AUC) and the glucose removal constant (KITT).

### 2.3 Histology

Liver samples were fixed in 10% formalin for 4 h and stored in 70% ethanol at 4 °C until being embedded in paraffin. Paraffin blocks were sectioned at 5 μm, stained with hematoxylin and eosin, and imaged using an optical microscope ICS Standard 25 (CarlZeiss, Brazil) with an AxioCam HRC (CarZeiss, Brazil) camera.

### 2.4 Western blotting

Liver samples were carefully homogenized in RIPA buffer supplemented with a protease inhibitor cocktail (Complete Ultra and Phospho-Stop, Roche, USA) and protein concentrations were determined by the Bradford assay (Bio-Rad®, Hercules, CA, USA). Total protein lysates (30 μg) were then separated by sodium dodecyl sulfate-polyacrylamide gel electrophoresis, and transferred to a nitrocellulose membrane. Membranes were incubated with antibodies against PPAR-γ (#2443) and TLR4 (#14358) purchased from Cell Signaling® (USA) and β-tubulin (#SC9109) purchased from Santa Cruz Biotechnology® (USA), followed by incubation with anti-IgG antibody conjugated with peroxidase. Following final incubation with peroxidase substrate (ECL kit, Biorad®, USA), immunoreactive protein bands were visualized/imaged (GBox Chemi, Syngene, USA), quantified by densitometry (ImageJ, 1.52p, National Institutes of Health, USA) and normalized by optical densitometry of bands incubated with β-tubulin.

### 2.5 Enzyme-linked immunosorbent assay (ELISA)

Protein was extracted from either liver or tissue fractions, as described above (Subsection 2.4) and concentrations of interleukin (IL)-1β, IL-6, monocyte chemoattractant protein (MCP)-1, and tumor necrosis factor (TNF)-α were determined by ELISA according to the manufacturer's instructions (DuoSet ELISA ®, R&D Systems, Minneapolis, MN, EUA).

### 2.6 Fractionation of liver cells

Hepatocytes and liver macrophages (LM) were isolated according to the method described previously by Zeng et al. [32]. Briefly, mice were subjected to a modified *in situ* liver perfusion technique [33,34], in which HBSS with HEPES (25 mM) followed by DMEM Low Glucose with HEPES (15 mM) and type IV collagenase were introduced into the portal vein by catheter. After perfusion, the liver was placed in a petri dish, minced, filtered, transferred to conical tubes and centrifuged (50 ×*g*, 2 min, 4 °C). Two fractions of the liver were obtained: parenchyma (pellet) and stroma (supernatant). The pellet containing the parenchymal fraction was washed twice with modified DMEM High Glucose (15 mM HEPES, 0.1 μM dexamethasone, 10% FBS) and purified hepatocytes were stored for further analysis. The supernatant containing cells of the stromal fraction was subjected to further centrifugation (800 ×*g*, 30 min, 25 °C, without brake) on a density gradient (Percol 25%, GE Healthcare, Sweden). The resulting pellet was then suspended in RPMI (10% FBS) and the cells obtained, mostly macrophages, were plated and incubated in RPMI (10% FBS, 1% penicilin/streptomicin) for 24 h (37 °C, 5% CO_2_) prior to flow cytometer analysis [32].

### 2.7 Flow cytometry

After 24 h incubation, LM were detached (PBS, 4 °C), centrifuged and re-plated (U bottom plate, 96 wells) for subsequent labeling with a conjugated antibody mix: F4/80-PerCP, CD11b-APC, CD11c-PECy7, CD86-FitC and CD206-PE (1: 100) (BD Biosciences, Franklin Lakes, NJ, USA). Beads incubated with each antibody were used as control compensators. Samples were analyzed on a FACSCanto II cytometer (BD Biosciences, Franklin Lakes, NJ, USA) using Diva-SofwareTM for data acquisition. 50,000 events per sample were acquired and FlowJo 10.0.7 software was used for data analysis.

### 2.8 RNA isolation, reverse transcription, and qRT-PCR

Total RNA was extracted with TRIZOL as described previously [35] and quantified in a spectrophotometer (NanoDrop® 2000; Thermo Fisher Scientific, Waltam, MA, EUA). The cDNA was synthesized from the total RNA using high-capacity cDNA Reverse Transcription Kit (Applied Biosystems, Foster City, CA, EUA). The sequences of the primers are shown in Table 1. Gene expression was quantified using QuantStudio 7 PCR System (Applied Biosystems, Foster City, CA, USA) and SYBER Green as a fluorescent marker. Gene expression of target genes was normalized by expression of B2M by the comparative CT method [36].

**Table 1:** Sequences of primers used for qRT-PCR.

Gene	NM	Primer Forward (5′- 3′)	Primer Reverse (3′ - 5′)
Arginase-1	007482.3	TCCTCCACGGGCAAATTCC	GCTGGACCATATTCCACTCCTA
B2M	009735.3	TTCTGGTGCTTGTCTCACTGA	CAGTATGTTCGGCTTCCCATTC
CCR2	009915.2	ATCCACGGCATACTATCAACATC	CAAGGCTCACCATCATCGTAG
CD106/VCAM	011693.3	AGTTGGGGATTCGGTTGTTCT	CCCCTCATTCCTTACCACCC
CD11c	021334.2	AGCCAGAGTGGGAAGTGAGA	AACTGGTCATGGCTGCTGA
CD36	001159555.1	ATGGGCTGTGATCGGAACTG	GTCTTCCCAATAAGCATGTCTCC
CD54/ICAM	010493.3	TGCCTCTGAAGCTCGGATATAC	TCTGTCGAACTCCTCAGTCAC
CD86	019388.3	TGTTTCCGTGGAGACGCAAG	TTGAGCCTTTGTAAATGGGCA
IDO-1	001293690.1	CATCACCATGGCGTATGTGT	CTCGCAGTAGGGAACAGCAA
MCP-1	011333.3	TAAAAACCTGGATCGGAACCAAA	GCATTAGCTTCAGATTTACGGGT
NFκB RelA	009045.4	AGGCTTCTGGGCCTTATGTG	TGCTTCTCTCGCCAGGAATAC
STAT6	009284.2	CTCTGTGGGGCCTAATTCA	CATCTGAACCGACCAGGAACT
TNFα	013693.3	TCTACTGAACTTCGGGGTGA	GATCTGAGTGTGAGGGTCTGG

### 2.9 Molecular modeling and molecular docking study of TRL-4/MD-2 complex

The 3D-structure of POA and its derivatives used this study was based on previous reports [37]. 3D-structures were optimized by the semi-empirical quantum chemical PM3 method used for partial charge calculation on the ligand by software Gaussian 09. The crystal structure of human MD-2 and its complex with anti-endotoxic lipid IVa (myristicacid; MA) was derived from the RCSB Protein Data Bank (http://www.rcsb.org/pdb/home/home.do). Protein structure was prepared for analysis by adding hydrogen atoms and fixing missing side-chains; missing loops were generated and sulfate/crystallization buffer molecules, such as glycerol, were removed using the biopolymer module implemented in the platform SYBYL 2.1 [38]. Amino acid residues were considered rigid and both structural water molecules were maintained in the active site. Finally, the polar hydrogens were added to the MD-2 model. Molecular docking was performed using the software GOLDv5 [39]. The docking runs were carried out with a radius of 10 Å, with coordinates by the MA binding pocket on MD-2. The best ranked docking pose of the ligands in the active site of MD-2 was determined according to the scores, binding-energy values and distance between the regions of hydrogen bonds (H-bonds), hydrophobic and phosphate interact. Docking poses within the binding site were visualized and figures were generated using PyMOL as previously described [40].

### 2.10 Statistical analysis

Normal distribution and variance homogeneity were tested and the appropriate statistical test (one-way analysis of variance [ANOVA] or two-way ANOVA) was employed followed by post-hoc testing (Bonferroni post-test). The data are presented as means ± standard error of the mean (SEM) and analyses were performed using GraphPad Prism 7.0 software. Differences were considered significant when *p* < 0.05.

## 3 Results

### 3.1 Palmitoleic acid improves whole-body insulin sensitivity in HFD-fed mice

**Fig. 1:**
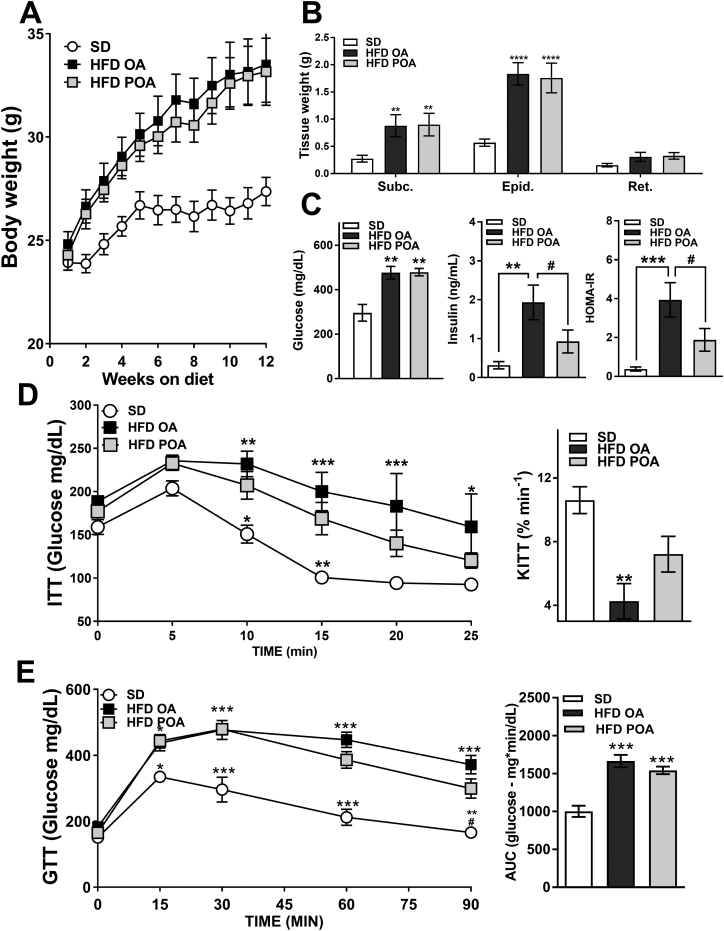
Palmitoleic acid restores basal and glucose-stimulated insulin sensitivity in HFD-fed mice. (A) Body weight change during high fat diet (HFD)-feeding (*n* = 10–12); (B) adipose depot weights (*n* = 5–7); (C) fasting glucose, insulin levels and HOMA-IR (n = 5–7); (D) glucose levels during insulin tolerance test (ITT) and respective glucose clearance constant (KITT) (n = 5–7); (E) glucose changes during glucose tolerance test (GTT) and respective area under curve (AUC) (n = 5–7). Wild-type (WT) mice fed with a standard diet (SD) or high-fat diet treated with oleic acid (HFD OA) or palmitoleic acid (HFD POA). Data are presented as the mean ± SEM. *p < 0.05, ^⁎⁎^*p* < 0.01, ^⁎⁎⁎^*p* < 0.001 ****p < 0.0001 *vs*. WT SD; ^#^p < 0.05 HFD POA *vs*. HFD OA. (One-way ANOVA followed by Bonferroni correction).

Supplementation with POA for 2 weeks did not change body weight gain compared OA supplementation in HFD fed mice (Fig. 1A), nor the tissue weights of white adipose depots (Fig. 1B). However, supplementation with POA did result in improved fasting HOMA-IR, and insulin levels compared with HFD-fed mice supplemented with OA (Fig. 1C) suggesting improved insulin sensitivity. POA-treated HFD-fed mice also exhibit improved insulin tolerance (Fig. 1D) and there was also a trend toward improved glucose tolerance at later time points, although AUC values did not reach statistical significance (Fig. 1E).

### 3.2 Palmitoleic acid promotes beneficial changes in liver of HFD-fed mice

**Fig. 2:**
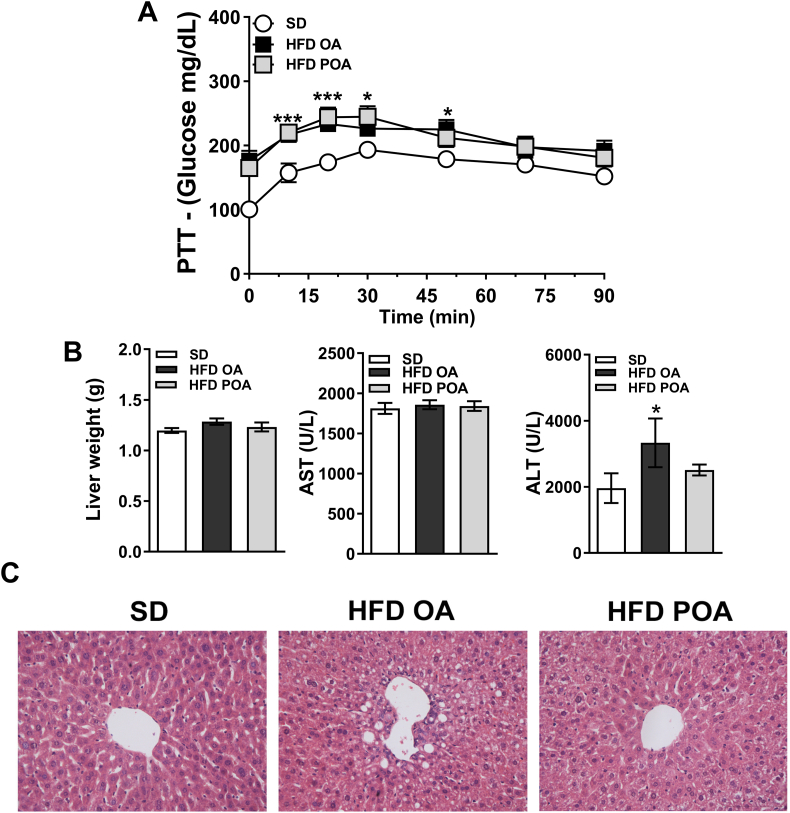
Palmitoleic acid reduces lipid accumulation and liver damage markers (ALT) in HFD-fed mice. (A) Glucose production on pyruvate tolerance test (PTT) (A) (*n* = 6–7); (B) liver weight and blood levels of aspartate transaminase (AST) and alanine aminostrasferase (ALT) (*n* = 4–6); (C) histological slices of livers stained with hematoxylin and eosin at 40 x magnification (representative of 4 mice per group). Wild-type (WT) mice fed with a standard diet (SD) or high-fat diet treated with oleic acid (HFD OA) or palmitoleic acid (HFD POA). Data are presented as the mean ± SEM. *p < 0.05, ^⁎⁎^p < 0.01, ^⁎⁎⁎^p < 0.001 *vs*. WT SD; ^#^p < 0.05 HFD POA *vs*. HFD OA. (One-way ANOVA followed by Bonferroni correction).

**Fig. S1:**
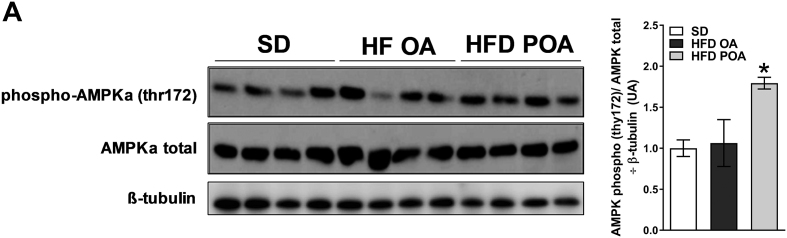
Palmitoleic acid induces AMPK activation. Liver phosphorylation of AMPK (p-thr172) normalized by the respective total protein (AMPK) and by β-tubulin (D). Wild-type (WT) mice fed with a standard diet (SD) or high-fat diet treated with oleic acid (HFD OA) or palmitoleic acid (HFD POA) (*n* = 4). Data are presented as the mean ± SEM. *p < 0.05, ^⁎⁎^*p* < 0.01, ^⁎⁎⁎^*p* < 0.001 *vs*. WT SD; ^#^p < 0.05 HFD POA *vs*. HFD OA. (One-way ANOVA followed by Bonferroni correction).

**Fig. S2:**
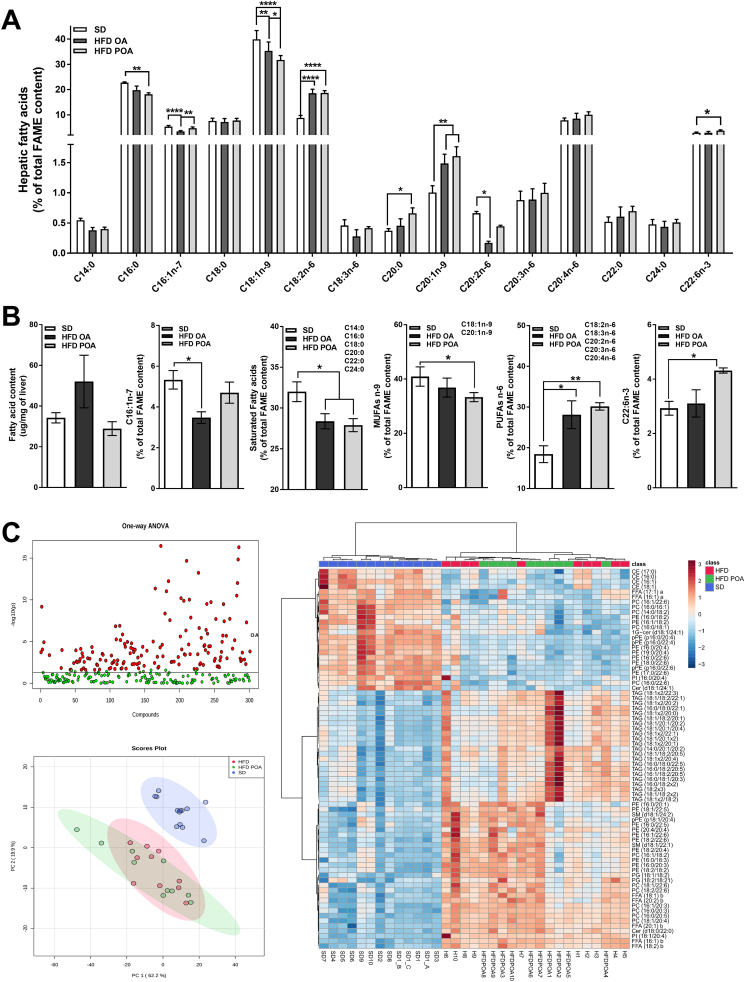
POA was successfully incorporated into liver but lipidomic does not show a clear segregation of the HFD-fed mice with or without POA treatment. Full panel of fatty acids species, measured by gas chromatography (GC), incorporated into the liver of wild-type (WT) mice fed with a standard diet (SD), high-fat fed mice treated with oleic acid (HFD OA) or palmitoleic acid (HFD POA) (A) (*n* = 5); liver content of total fatty acids (μm/mg of liver) (first graph), palmitoleic acid (C16:1,n-7) (second graph), total lipid species of saturated acid (third graph), total lipid species of monounsaturated n-9 fatty acids (MUFA n-9) (fourth graph), total lipid species of polyunsaturated n-6 fatty acids (PUFA n-6) (fifth graph), and Docosahexaenoic acid (C22:6, n-3) (sixth) estimated by GC (B) (n = 5); full panel of liver lipid species obtained by lipidomic (C) (n = 5). The data are presented as the mean ± SEM (n = 5). *p < 0.05, ^⁎⁎^*p* < 0.01, ^⁎⁎⁎^*p* < 0.001 ****p < 0.0001 *vs*. indicated groups. (One-way ANOVA followed by Bonferroni correction).

POA did not change elevated hepatic gluconeogenesis in HFD-fed mice, as determined by the PTT (Fig. 2A). However, POA-treated HFD-fed mice exhibited lower levels of the liver-damage marker alanine aminotransferase (ALT) (Fig. 2B), less liver steatosis (Fig. 2C), and increased hepatic activation of the anti-inflammatory and pro-oxidative metabolic sensor, AMPK, compared to OA-treated HFD-fed mice (Fig. S1). Gas chromatography analysis showed that POA (C16:1n-7) in the liver was increased to the same proportion as in SD group (Fig. S2A). The lipidomic data also pointed to a clear separation of liver samples extracted from SD-fed mice *versus* HFD OA-fed mice, displaying 75 altered lipid species (Fig. S2C). However, a clear segregation of the HFD-fed mice with OA or POA treatment was not observed with principal component analysis (Fig. S2C).

### 3.3 Palmitoleic acid promotes anti-inflammatory effects in liver of HFD-fed mice and modulates liver macrophage populations

**Fig. 3:**
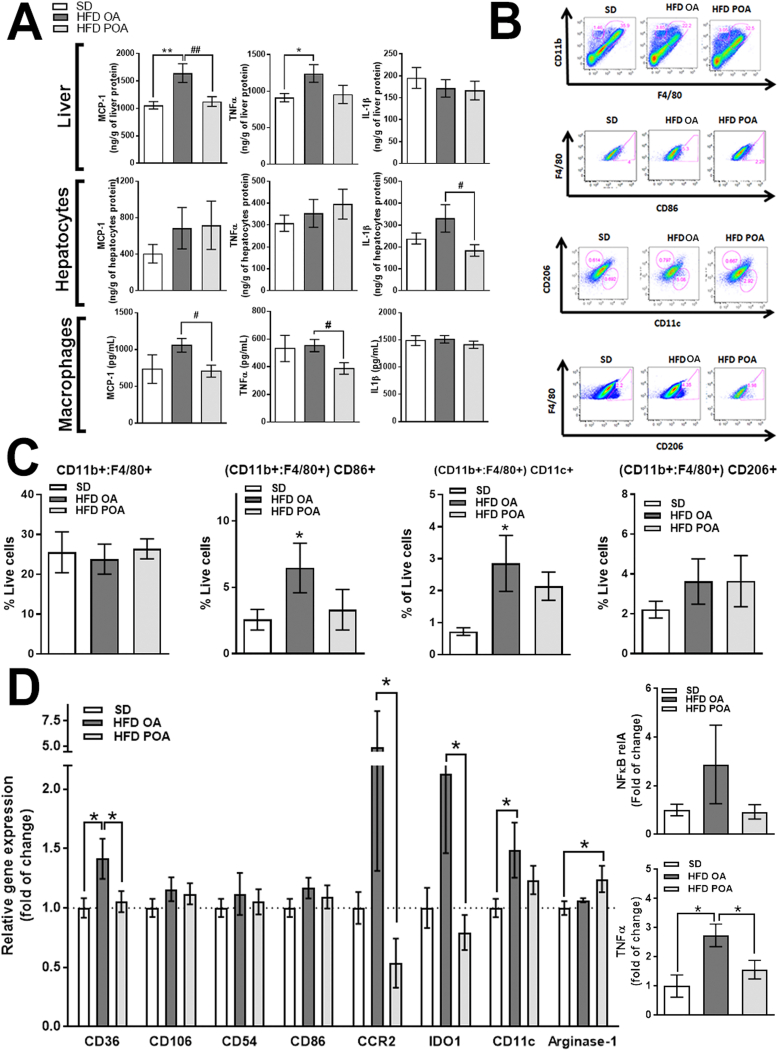
Palmitoleic acid promotes anti-inflammatory effects in liver of HFD-fed mice by modulation of the liver macrophage population. (A) Monocyte chemoattractant protein (MCP)-1, interleukin (IL)-1β and tumor necrosis factor (TNF)-α levels in liver, hepatocytes and liver macrophages (LM) (*n* = 11–15); (B) flow cytometry gate strategy (representative of n = 4–5); (C) % of F4/80, CD11c, CD86 and CD206 positive LM (n = 4–5), (D) relative mRNA expression of inflammation-related genes in LM (n = 5–7). Wild-type (WT) mice fed with a standard diet (SD) or high-fat diet treated with oleic acid (HFD OA) or palmitoleic acid (HFD POA). Ct were normalized to B2M. The data are presented as the mean ± SEM. *p < 0.05 *vs*. indicated groups. (One-way ANOVA followed by Bonferroni correction).

Consistent with the lower ALT levels, less hepatic steatosis, and higher AMPK activity, POA treatment reduced hepatic levels of the pro-inflammatory cytokines MCP-1 and TNF-α compared with OA treatment, while IL-1β remained unchanged (Fig. 3A). Interestingly, tissue fractionation revealed cell population-specific effects. POA treatment reduced the levels of IL-1β in hepatocytes compared to OA treatment. However, similar to whole liver, POA supplementation reduced MCP-1 and TNF-α levels in liver macrophages (LM), compared with OA treatment (Fig. 3A). This strongly suggests that POA modulates the LM population; therefore, we next isolated and profiled the LM population.

The total number of myeloid-derived macrophages (CD11b(hi) and F4/80(hi)) was unaltered, however, there was a greater proportion of CD86^+^ and CD11c^+^ macrophages in liver of HFD OA mice, which tended to be reduced by POA treatment (Fig. 3B, C). Similarly, LM of POA treated HFD-fed mice showed a significant anti-inflammatory phenotype, with reductions in the levels of mRNA for the pro-inflammatory markers, CD36, CCR2, indoleamine 2,3-dioxygenase (IDO)-1 and TNF-α compared with OA treatment, and increased mRNA levels of the anti-inflammatory marker, arginase-1, compared with the SD group (Fig. 3D).

### 3.4 Palmitoleic acid increases PPAR-γ in liver

**Fig. 4:**
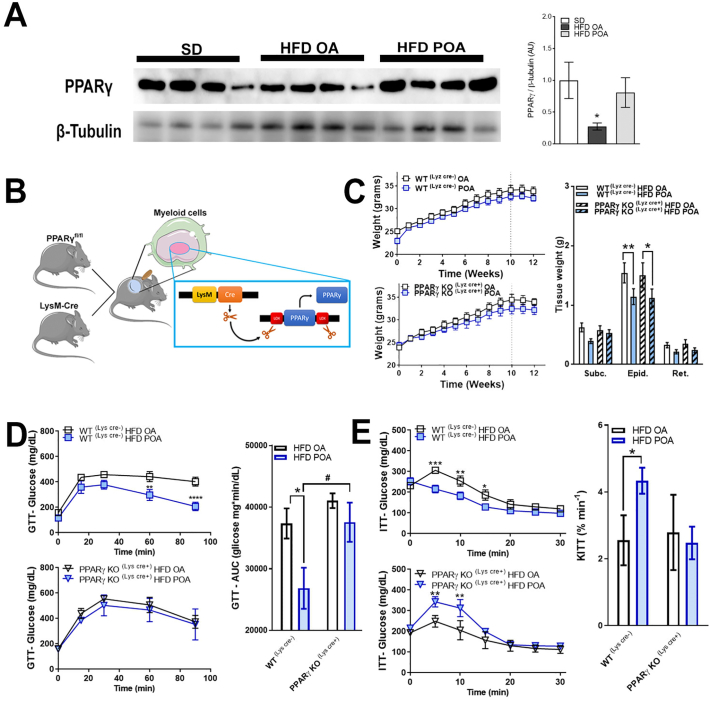
Palmitoleic acid increases PPAR-γ in liver and restores HFD-related diabetes only in WT mice, not in macrophage-specific PPAR-γ KO mice. (A) liver protein levels of peroxisome proliferator activated receptor gamma (PPAR-γ) normalized by the respective β-tubulin (n = 4); (B) scheme for generation of specific myeloid cell PPAR-γ knockout mice showing that PPAR-γ Flox mice were crossed with Lysozyme M-Cre (LysCre) mice; (C) body weight change during high fat diet (HFD) feeding (*n* = 7–10) and adipose tissue weight (n = 7–10); (D) glucose levels on glucose tolerance test (GTT) and respective area under curve (AUC) (n = 4–5); (E) glucose levels during insulin tolerance test (ITT) and respective glucose clearance constant (KITT) (*n* = 3–5). Wild-type (WT) mice fed with a standard diet (SD) or high-fat diet treated with oleic acid (HFD OA) or palmitoleic acid (HFD POA) (A), WT(Cre-) or PPAR-γ KO (Cre+) mice fed with high-fat diet and treated with oleic acid (HFD OA) or palmitoleic acid (HFD POA) (C, D and E). Data are presented as the mean ± SEM. *p < 0.05 *vs*. indicated groups. (One-way ANOVA (A) or two-way ANOVA (C, D and E) followed by Bonferroni correction).

**Fig. S3:**
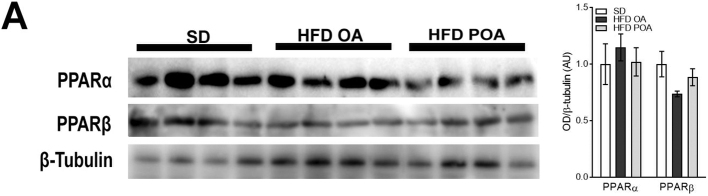
Palmitoleic acid does not change PPAR-α and PPARβ expression in liver of HFD-fed WT mice (A) liver protein levels of peroxisome proliferator activated receptor (PPAR)-α and PPAR-β normalized by the respective β-tubulin levels (*n* = 4). Data are presented as the mean ± SEM. *p < 0.05 *vs*. indicated groups. (One-way ANOVA followed by Bonferroni correction).

Livers of POA-treated HFD-fed mice exhibited higher levels of the lipogenic, anti-inflammatory and phenotypic switching transcription factor, PPAR-γ compared with livers of OA-treated mice (Fig. 4A). However, POA treatment did not change the hepatic levels of other PPARs (PPAR-α or PPAR-β) (Fig. S3). Since PPAR-γ is implicated in the anti-inflammatory polarization of macrophages, we next investigated whether the beneficial anti-inflammatory effect of POA in liver macrophages of HFD-fed mice is dependent on PPAR-γ. To do this, we crossed the PPAR-γ floxed (PPARγ^fl/fl^) mice with lysozyme M-Cre (LysCre^+ve^) mice to generate, PPAR-γ KO (LysCre^+^) mice which lack PPARγ specifically in myeloid cells (Fig. 4B).

### 3.5 Palmitoleic acid does not reverse HFD-related diabetes in myeloid-specific PPAR-γ KO mice

The selective deletion of PPAR-γ in macrophages and the POA treatment did not alter the body weight gain induced by HFD; however, independent of the genotype, POA-treatment reduced epididymal fat weight (Fig. 4C). POA treatment significantly improved both glucose tolerance and insulin sensitivity in HFD-fed WT(LysCre-) mice. However, POA failed to improve glucose tolerance (Fig. 4D) and insulin resistance (Fig. 4E) in myeloid-specific PPAR-γ KO (LysCre^+^) mice fed a HFD diet.

### 3.6 Palmitoleic acid reduces HFD-induced liver inflammation by modulation of liver macrophages in myeloid-specific PPAR-γ KO mice

**Fig. 5:**
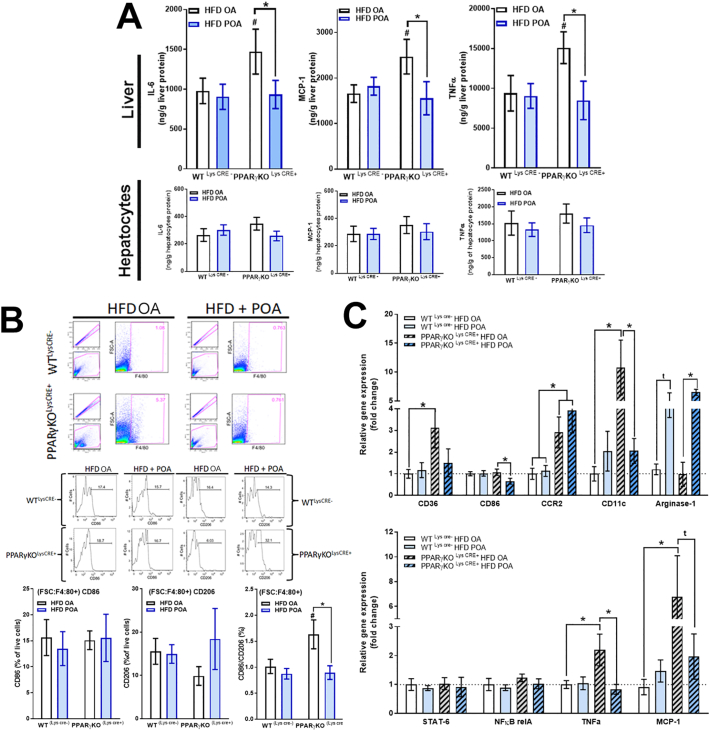
Palmitoleic acid reduces liver inflammation by modulation of the liver macrophages population and independently of macrophage-specific PPAR-γ knockout. (A) Interleukin (IL)-6, monocyte chemoattractant protein (MCP)-1 and tumor necrosis factor (TNF)-α levels in liver and hepatocytes (n = 6–10); (B) flow cytometry gate strategy and % of F4/80, CD86 and CD206 positive liver macrophages (LM) (n = 3–5); (C) relative mRNA expression of inflammation-related genes in LM normalized by B2M (n = 3–6). Wild-type (WT) (Cre-) or PPAR-γ KO (Cre+) mice fed with high-fat diet treated with oleic acid (HFD OA) or palmitoleic acid (HFD POA). Data are presented as the mean ± SEM. *p < 0.05 *vs*. indicated groups. (Two-way ANOVA followed by Bonferroni correction).

In myeloid-specific PPAR-γ KO (LysCre^+^) mice fed HFD, POA treatment reduced the hepatic expression of MCP-1, IL-6 and TNF-α, but this was not reflected in isolated hepatocytes from the same mice (Fig. 5A). Similarly, POA reduced the CD86/CD206 ratio in the liver macrophage population isolated from PPAR-γ KO mice (LysCre+) (Fig. 5B). In addition, LM of POA treated PPAR-γ KO (LysCre+) mice also exhibited a profound anti-inflammatory phenotype, with downregulated gene expression of pro-inflammatory macrophage markers such CD36, CD86, CD11c, MCP-1 and TNF-α, and upregulated gene expression of arginase-1 (anti-inflammatory M2 marker) (Fig. 5C).

Therefore, we show that the anti-inflammatory effects of POA in liver are mediated by modulation of LM. However, PPAR-γ activation in myeloid cells is apparently not required for this effect.

### 3.7 Palmitoleic acid reduces TLR4 activation in liver of HFD-fed mice and structural prediction model indicates palmitoylation plays a role in this interaction

**Fig. 6:**
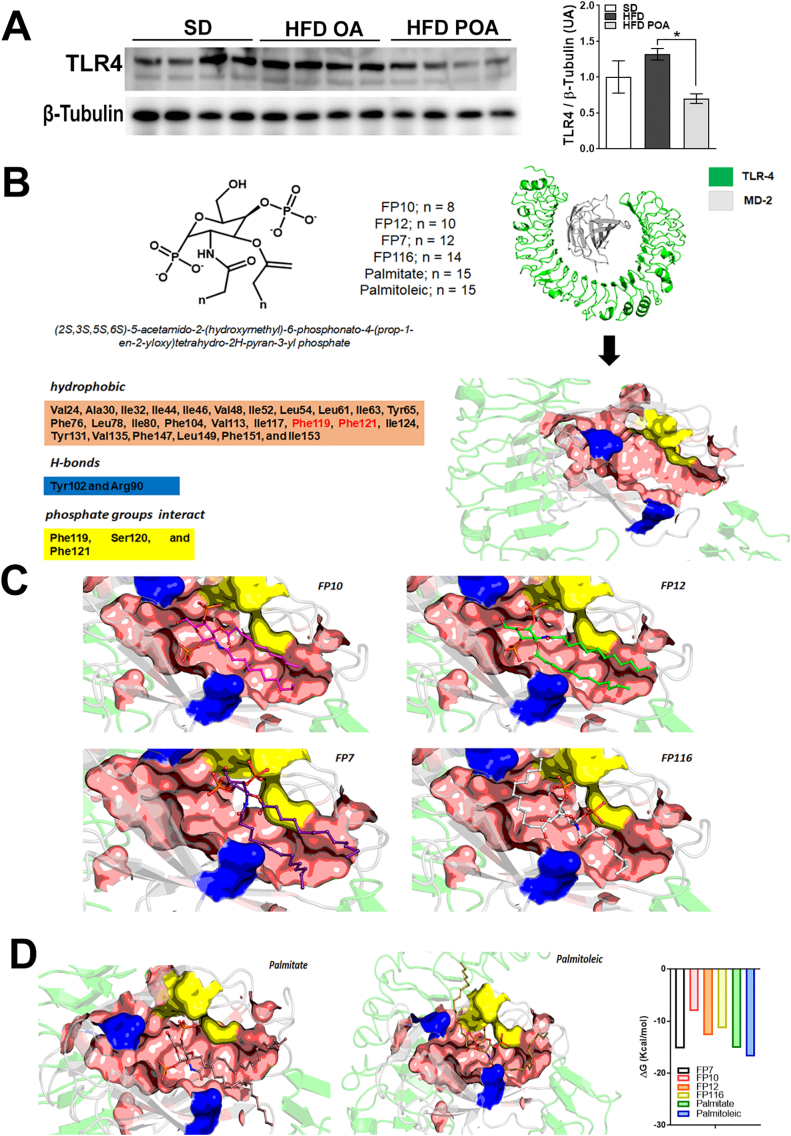
Palmitoleic acid reduces TLR4 activation in liver of HFD-fed mice and the structural prediction model indicates palmitoylation plays a role in that interaction. (A) hepatic protein levels of toll-like receptor (TLR)-4 normalized by the respective β-tubulin (n = 4); (B) Docking analysis of TRL-4/MD-2 complex with (9Z)-hexadec-9-enoic acid and derivates; (C and D) energy of binding (ΔG) to the disruptive potential of different fatty acids and TLR-4 antagonist to block TLR4-MD2 dimer formation and interaction. Livers of WT mice fed with a standard diet (SD) or high-fat diet treated with oleic acid (HFD OA) or palmitoleic acid (HFD POA). Data are presented as the mean ± SEM. ^#^p < 0.05 *vs*. indicated groups. (One-way ANOVA followed by Bonferroni correction).

Our previous study showed POA reduced expression of TLR4 in liver [12]. To explore this target in the PPARg-independent actions of POA, we investigated TLR4 expression in the current study. In accordance with our previous findings, the protein levels of TLR4 in liver of POA-treated HFD-fed mice were reduced compared with OA-treated obese mice (Fig. 6A). However, by generating a protein-protein docking computational model, we predict that palmitate and POA bind to affect the TLR4-MD2 complex in opposite ways. Palmitate is likely to engage with the interaction regions of MD2, and does not disturb the TLR4-MD2 complex, whereas POA's unsaturation allows it to interact directly with TLR-4, disrupting the TLR4-MD2 complex (Fig. 6B). This disturbance of the complex creates a low ΔG value, similar to what is observed for FP7 (antagonist of TLR4-MD2) interaction (Fig. 6B).

## 4 Discussion

Our major findings are that POA supplementation for 2 weeks is sufficient to reduce the development of HFD-induced insulin resistance and liver inflammation, reducing lipid storage in the parenchyma. The molecular mechanism by which this may occur seems to involve a decrease in CD36 mRNA and a proportional increase in the anti-inflammatory/proinflammatory macrophage ratio independent of PPAR-γ. Moreover, this effect of POA on hepatic inflammation is closely related to the pattern of cytokines produced by macrophages, but not hepatocytes. Finally, deletion of PPAR-γ in myeloid cells did not prevent the anti-inflammatory effects of POA supplementation, but it did prevent POA-induced improvements in glucose and insulin tolerance.

POA has been described as an insulin-sensitizing molecule (11). Consistent with this, HFD-fed mice supplemented with POA exhibited better insulin and glucose tolerances. This beneficial effect of POA was absent in myeloid-specific PPAR-γ KO mice. Consistent with our results, mice lacking PPAR-γ in myeloid cells also exhibit impaired insulin signaling in liver, adipose tissue and skeletal muscle [41]. This is thought to occur because PPAR-γ depletion in macrophages reduces its ability to polarize toward the anti-inflammatory state [41,42].

**Fig. S4:**
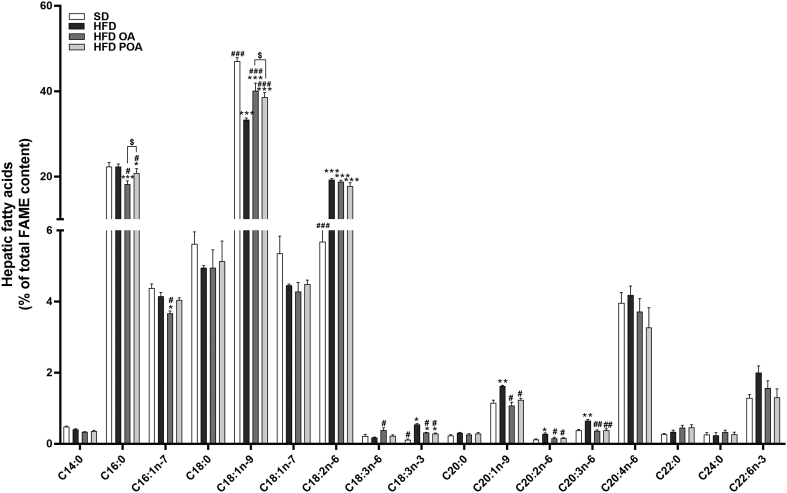
Liver of mice fed with a HFD without daily gavage of OA or POA exhibited higher levels of palmitic (C16:0) and lower oleic acid (C18:1n-9). Full panel of fatty acids species, measured by gas chromatography (GC), incorporated into the liver of wild-type (WT) mice fed with a standard diet (SD), high-fat fed mice without treatment (HFD), treated with oleic acid (HFD OA) or palmitoleic acid (HFD POA) (A) (n = 4–5). The data are presented as the mean ± SEM. *p < 0.05, ^⁎⁎^p < 0.01, ^⁎⁎⁎^p < 0.001 groups *vs*. SD; ^#^p < 0.05, ^##^p < 0.01, ^###^p < 0.001 groups *vs*. HFD, ^$^p < 0.05, HFD OA *vs*. HFD POA. (One-way ANOVA followed by Bonferroni correction).

POA administration promoted the increase of POA in the liver FFAs, reducing lipid storage, pro-inflammatory markers, and serum ALT, and increasing AMPK phosphorylation. These effects are not related with the major lipid species in the liver, since we have not observed major differences between the two HFD groups with regard to the lipid species present. However, it is important to consider that the control group (HFD OA) received a daily gavage of OA, which although not significant when compared to the total amount of OA found in the diet (Table S2), does induce some changes in the liver fatty acid content (Fig. S4).

Inflammation plays a central role in the pathogenesis of NAFLD to NASH and in the aggravation of the steatosis and development of cirrhosis and hepatocellular carcinoma [6]. Liver inflammation can be mediated locally, resulting from cellular stress increased by lipid accumulation in hepatocytes (lipotoxicity). This cellular lipotoxicity can trigger mitochondrial and endoplasmic reticulum stress responses and activate immune cells (Kupffer cells, non-resident macrophages and neutrophils) [6,43]. Additionally, liver inflammation can also be triggered by a systemic response. For instance, obesity-related inflammation is associated with systemic gut dysbiosis that might increase blood endotoxin levels and activate toll like receptor (TLR)4 in tissue-resident macrophages, triggering the pro-inflammatory cascade and increasing chemoattractant chemokines and promoting immune-cell migration into specific organs, such as the liver [44].

Our previous studies described that POA promoted anti-inflammatory effects in the liver of HFD-fed mice [12] and in LPS-stimulated cultured macrophages [22]. Therefore, we isolated hepatic macrophages and hepatocytes to analyze the anti-inflammatory effect of POA supplementation *in vivo*. High fat diet feeding increased pro-inflammatory cytokines in both cell types, but in a cell-specific manner. In hepatocytes, IL-1β was increased by the HFD and POA reduced this to levels seen in the SD group. POA reduced MCP-1 and TNF-α protein in LM. Interestingly, the profile of cytokines in the whole liver is similar to that in LM. The HFD increased the proinflammatory CD86^+^ macrophages in the liver and POA supplementation was able to reduce this subset of macrophages.

In this study, HFD reduced the liver protein levels of PPAR-γ, which were restored with POA supplementation. In liver, PPAR-γ exhibits a dual function: it can induce lipogenic genes and favor lipid accumulation, while at same time it promotes anti-inflammatory effects because of its role as a NFκB trans-repressor [[45], [46], [47]]. As an example of this dualism, liver of exercise trained HFD-fed PPARα KO mice exhibited lower PPAR-γ expression and less hepatic lipid accumulation, but higher levels of pro-inflammatory cytokines [48]. On the other hand, when treated with rosiglitazone (a PPAR-γ agonist), the liver-expression of PPAR-γ is restored in these trained HFD-fed PPARα KO mice although they still have exhibited lower hepatic inflammation [48]. One can speculate that PPARγ agonists could exhibit a cumulative anti-inflammatory effect with POA treatment, which would be of great interest and should be the subject of future investigations.

In macrophages, PPAR-γ triggers polarization to an anti-inflammatory phenotype, reducing inflammation [49,50]. Our previous study showed that POA supplementation increased the expression of PPAR-γ in cultured primary macrophages (12). The lack of PPAR-γ in myeloid cells increased the levels of pro-inflammatory cytokines (IL-6, TNF-α and MCP-1) only in the liver, but not hepatocytes. Similarly, liver macrophages from these mice also exhibited high mRNA levels of TNF-α and MCP-1, as well as macrophage pro-inflammatory markers, such CCR2 and CD11c, and a higher CD86(M1)/CD206(M2) population ratio. However, contrary to our hypothesis, POA treatment restored most of these inflammation-related abnormalities in these myeloid PPAR-γ KO mice. POA treatment reduced liver cytokine levels, the mRNA of TNF-α, MCP-1 and CD11c, and also increased the CD206^+^ (anti-inflammatory) population, therefore reducing the ratio of pro-inflammatory/anti-inflammatory macrophages. These results indicate that the effect of POA upon inflammatory pathways is independent of PPAR-γ expression in liver macrophages.

In fact, POA treatment can have anti-inflammatory effects independently of PPAR-γ. For instance, our previous findings indicated that POA blocked the inflammatory cascade triggered by endotoxin, by reducing TLR-4 and NFκB expression, and by decreasing the production of pro-inflammatory cytokines in cultured-primary macrophages [22]. Therefore, it is suggestive that POA treatment promotes an anti-inflammatory effect in livers of HFD-fed mice, largely due to modulation of the phenotype of liver-macrophages, while also increasing hepatic PPAR-γ levels.

POA supplementation reduced the higher expression of CD36 exhibited by macrophages from HFD-fed mice. The expression of CD36 in LM was increased by HFD-feeding in WT and in macrophage-specific PPAR-γ KO mice. Similarly, independently of PPAR-γ, mice supplemented with POA showed a reduction in CD36 expression. Recently, Zhao et al. demonstrated that patients with non-alcoholic steatohepatitis had higher expression of CD36, such a phenotype that was linked to an increase in liver inflammation and fibrosis. Furthermore, by increasing liver fatty acid efflux, CD36 higher expression has been suggested to reduce AMPK activity and therefore fatty acid oxidation [51]. Thus, the reduction in CD36 induced by POA can perhaps mediate the decrease inflammation and increase AMPK activity, seen here and in our previous study [21].

Our previous studies also showed a HFD-induced increased in hepatic TLR4 expression, while POA supplementation prevented this increase [12] (Fig. 6A). The activation of TLR4 is dependent on the heterodimerization with co-receptor, MD-2. This TLR4-MD2 dimer complex is stabilized in the hydrophobic region, the F126 loop. An initial step in the canonical activation pathway is dimerization with other TLR4-MD2 dimers (dimer of dimers), in an MYD-88 dependent mechanism [52]. Here our computational modeling suggests that POA results in a lower ΔG for the interaction with MD2, similar to that of the TLR4 antagonist, FP7, which is known to interact in the same F126 loop site.

Taken together, our results show that in HFD-fed mice, POA supplementation improves whole body glucose homeostasis, promotes an important anti-inflammatory effect in the liver, and decreases hepatic lipid accumulation possibly due to lower CD36 expression and higher AMPK phosphorylation. In addition, our results shed light on the molecular mechanisms by which POA acts upon liver immune-metabolic disturbances induced by HFD feeding. We have demonstrated that in HFD-fed mice, POA markedly lowers the inflammatory cytokines produced by LM. POA intake also reduced the proinflammatory subset in HFD-fed WT mice, while increasing PPAR-γ expression. Following that, we questioned whether the anti-inflammatory role of POA would still occur in HFD-fed mice that lacked PPAR-γ in myeloid cells. Interestingly, POA lowered pro-inflammatory cytokines, reduced inflammation-related genes, including CD36, and induced similar changes in the macrophage population profile, independently of PPAR-γ.

In conclusion, supplementation with POA induces a number of beneficial effects in liver metabolism and inflammation. POA treatment may protect the liver from lipid accumulation due to an increase of fatty acid oxidation (*via* AMPK phosphorylation) and a decrease of fatty acid uptake (*via* a reduction in CD36). POA can control the fatty liver-related inflammation by induction of anti-inflammatory macrophages independently of PPAR-γ.

The following are the supplementary data related to this article.

Table S1 Standard diet (SD) and High Fat Diet (HFD) composition. Table S1

Table S2 Standard diet (SD) and High Fat Diet (HFD) fatty acid composition. Table S2

Supplementary material Image 1

## CRediT authorship contribution statement

**Camila O. Souza:** Investigation, Formal analysis, Writing - original draft. **Alexandre A.S. Teixeira:** Investigation, Formal analysis. **Luana Amorim Biondo:** Investigation, Formal analysis. **Loreana Sanches Silveira:** Investigation, Formal analysis. **Cristiane N. de Souza Breda:** Investigation, Formal analysis. **Tarcio T. Braga:** Investigation, Formal analysis. **Niels O.S. Camara:** Data curation, Formal analysis, Methodology, Writing - review & editing. **Thiago Belchior:** Investigation, Formal analysis. **William T. Festuccia:** Data curation, Formal analysis, Methodology, Writing - review & editing. **Tiego A. Diniz:** Investigation, Formal analysis. **Glaucio Monteiro Ferreira:** Investigation, Formal analysis. **Mario Hiroyuki Hirata:** Data curation, Formal analysis, Methodology, Writing - review & editing. **Adriano B. Chaves-Filho:** Investigation, Formal analysis. **Marcos Y. Yoshinaga:** Data curation, Formal analysis, Methodology, Writing - review & editing. **Sayuri Miyamoto:** Data curation, Formal analysis, Methodology, Writing - review & editing. **Philip C. Calder:** Formal analysis, Writing - review & editing. **Jaswinder K. Sethi:** Formal analysis, Writing - review & editing. **José C. Rosa Neto:** Supervision, Formal analysis, Writing - original draft, Writing - review & editing.

## Declaration of competing interest

The authors declare that they have no known competing financial interests or personal relationships that could have appeared to influence the work reported in this paper.
