# Supplementary material for: Palmitoleic acid reduces high fat diet-induced liver inflammation by promoting PPAR-γ-independent M2a polarization of myeloid cells
Source: Biochim Biophys Acta Mol Cell Biol Lipids. 2020 Oct;1865(10):158776. doi: 10.1016/j.bbalip.2020.158776 (PMC7487782; doi:10.1016/j.bbalip.2020.158776)
Supplement: Table S1 — Standard diet (SD) and High Fat Diet (HFD) composition. [file mmc5.docx]

Table S1 – Standard diet (SD) and High Fat Diet (HFD) composition.

| Ingredients  (g/Kg) | SD  (10% fat diet) | HFD  (60% fat diet) |
| --- | --- | --- |
| Choline bitartrate (g) | 2.5 | 2.5 |
| L-Cystine (g) | 1.8 | 1.8 |
| Vitamin Mix (g) | 10 | 10 |
| Mineral Mix (g) | 35 | 35 |
| Cellulose (g) | 50 | 50 |
| Sucrose (g) | 100 | 223.75 |
| Corn starch (g) | 467.5 | 57.75 |
| Maltodextrin (g) | 155 | 66 |
| Casein (g) | 140 | 200 |
| Hydrogenated vegetable fat (g) | 0 | 315 |
| Soybean oil (g) | 40 | 0 |
| Sunflower oil (g) | 0 | 40 |
