# Supplementary material for: Palmitoleic acid reduces high fat diet-induced liver inflammation by promoting PPAR-γ-independent M2a polarization of myeloid cells
Source: Biochim Biophys Acta Mol Cell Biol Lipids. 2020 Oct;1865(10):158776. doi: 10.1016/j.bbalip.2020.158776 (PMC7487782; doi:10.1016/j.bbalip.2020.158776)
Supplement: Table S2 — Standard diet (SD) and High Fat Diet (HFD) fatty acid composition. [file mmc6.docx]

Table S2 – Standard diet (SD) and High Fat Diet (HFD) fatty acid composition.

| Fatty acids | Fatty acid concentration (μg/mg of feed) | |
| --- | --- | --- |
|  | SD | HFD |
| C8:0 | **nd** | **nd** |
| C10:0 | **0.37 ± 0.7** | **nd** |
| C11:0 | **nd** | **nd** |
| C12:0 | **nd** | **1.8 ± 0.5** |
| C13:0 | **nd** | **0.2 ± 0.4** |
| C14:0 | **nd** | **1.1 ± 0.3** |
| C14:1n-5 | **nd** | **nd** |
| C15:0 | **nd** | **nd** |
| C15:1n-5 | **nd** | **nd** |
| C16:0 | **4.2 ± 0.8** | **40.5 ± 1.4** |
| C16:1n-7 | **nd** | **0.2 ± 0.1** |
| C17:0 | **4.4 ±0.8** | **4.4 ± 0.2** |
| C17:1n-7 | **nd** | **nd** |
| C18:0 | **1.2 ± 0.4** | **33.8 ± 0.7** |
| C18:1n-9 | **7.8 ± 1.2** | **200.7 ± 2.7** |
| C18:2n-6 | **17.3 ± 1.9** | **37.0 ± 1.9** |
| C18:3n-6 | **nd** | **0.3 ± 0.0** |
| C18:3n-3 | **1.3 ± 0.5** | **0.3 ± 0.0** |
| C20:0 | **nd** | **1.3 ± 0.1** |
| C20:1n-9 | **nd** | **0.7 ± 0.0** |
| C20:2n-6 | **nd** | **nd** |
| C20:3n-6 | **nd** | **nd** |
| C21:0 | **nd** | **nd** |
| C20:4n-6 | **nd** | **nd** |
| C20:3n-3 | **nd** | **nd** |
| C20:5n-3 | **nd** | **nd** |
| C22:0 | **0.1 ± 0.2** | **1.6 ± 0.2** |
| C22:1n-9 | **nd** | **nd** |
| C22:2n-6 | **nd** | **nd** |
| C23:0 | **nd** | **0.1 ± 0.0** |
| C24:0 | **nd** | **0.2 ± 0.3** |
| C22:6n-3 | **nd** | **nd** |
| C24:1n-9 | **nd** | **nd** |

nd: not detected
