# Supplementary material for: Palmitoleic acid reduces high fat diet-induced liver inflammation by promoting PPAR-γ-independent M2a polarization of myeloid cells
Source: Biochim Biophys Acta Mol Cell Biol Lipids. 2020 Oct;1865(10):158776. doi: 10.1016/j.bbalip.2020.158776 (PMC7487782; doi:10.1016/j.bbalip.2020.158776)
Supplement: Supplementary file 3 — Supplementary material [file mmc7.docx]

**Supplementary Material and Methods.**

**Western blotting**

Procedure was performed as described in section 2.4. Membranes were incubated with antibodies against phospho AMPKα (Thr 172, #2535), total AMPK (#2603) purchased from Cell Signaling® (USA) or PPAR-α (#SC9000), PPAR-β (#SC7197) and β-tubulin (#SC9109), purchased from Santa Cruz Biotechnology® (USA).

**Gas chromatography**

The fatty acid composition of liver samples was evaluated by gas chromatography (GC) of fatty acid methyl esters (FAMEs) as previously described [38, 39]. Liver (100 mg) was homogenized in ice with a tissue grinder in 1 mL of 10 mM phosphate buffer (pH 7.4) containing deferoxamine mesylate (100 μM). Briefly, 100 μL of liver homogenate was mixed with methanol (1.75 mL), acetyl chloride (100 μL) and internal standard solution containing 1 mg/mL heptadecanoic acid (50 μL). Samples were heated for 60 min at 100 ^o^C and then cooled in an ice bath. FAMEs were extracted using hexane (1.75 mL) with vigorous vortexing for 1 min and centrifugation at 1500 × g, 4 ^o^C for 2 min. After centrifugation, the upper organic phase was collected, evaporated under nitrogen and then dissolved in hexane (100 μL). The individual FAMEs were analyzed by GC with flame ionization detection. GC analysis was performed on a Trace 1310 (Thermo Scientific) using a DB-FFAP column of 15 m x 0.1 mm ID x 0.1 μm film thickness (J and W Scientific from Agilent Technologies). The temperature program started with an initial temperature of 150 ºC with a 0.25 min hold, which was increased at a rate of 35 ºC/min to 200 ºC, and then at 8 ºC/min to 250 ºC. Additional instrument conditions were: hydrogen/nitrogen was used as carrier gas at constant pressure of 345 kPa; FID temperature set at 250 ºC; air and nitrogen make-up gas flow were 350 mL/min and 40 mL/min; split ratio was 30:1; autosampler injections were 1 μL. Run time for a single sample was 12 min. FAMEs were identified by direct comparison with a FAME standard mix (Supelco 37 Component FAME Mix; Sigma-Aldrich); each individual peak was integrated and then normalized according to the peak of the internal standard. The percentage of individual FAME was made in relation to total area of FAME peaks.

**Lipidomic (intact lipid profile) analysis**

Non-targeted lipidomic analysis of liver was performed by liquid chromatography coupled to mass spectrometry (LC-MS) as previously described [40]. Briefly, 100 µL of liver homogenate (as described for GC analysis session) was mixed with 400 μL of phosphate buffer, 400 μL of ice-cold methanol and 100 μL of internal standards. Next, 1.5 mL of chloroform/ethyl acetate (4:1) was added to the mixture, which was thoroughly vortexed for 30 s. After centrifugation at 1500 x g for 2 min at 4 °C, the lower phase containing the total lipid extract (TLE) was transferred to a new tube and dried under N_2_ gas. Dried TLE were redissolved in 100 μL of isopropanol and the injection volume was set at 1 µL. Blanks and quality controls were injected every 5 and 10 samples, respectively.

TLEs were analyzed by ESI-Q-TOFMS (Triple TOF 6600, Sciex, Concord, US) interfaced with a high-performance LC (UHPLC Nexera, Shimadzu, Kyoto, Japan). The samples were loaded into a CORTECS (UPLC C18 column, 1.6 µm, 2.1 mm i.d. × 100 mm) with a flow rate of 0.2 mL/min and the oven temperature was maintained at 35 °C. Mobile phases A (water:acetonitrile, 60:40) and B (isopropanol:acetonitrile:water, 88:10:2) contained ammonium acetate or ammonium formate (at a final concentration of 10 mM) for experiments performed in negative or positive ionization mode, respectively. Lipids were separated by a 20 min linear gradient as follows: from 40 to 100% B over the first 10 min., hold at 100% B from 10–12 min., decreased from 100 to 40% B during 12–13 min., and hold at 40% B from 13–20 min.

The MS was operated in both positive and negative ionization modes, and the scan range set at a mass-to-charge ratio of 200–2000 Da. Data for lipid molecular species identification and quantification were obtained by Information Dependent Acquisition (IDA^®^). Data acquisition using Analyst® 1.7.1 was performed with a cycle time period of 1.05 s with 100 ms acquisition time for MS1 scan and 25 ms acquisition time to obtain the top 36 precursor ions. An ion spray voltage of −4.5 kV and 5.5 kV (for negative and positive modes, respectively) and the cone voltage at +/−80 V were set to analysis. Additional parameters included curtain gas set at 25 psi, nebulizer and heater gases at 45 psi and interface heater of 450 °C. The MS/MS data were analyzed with PeakView, and lipid molecular species were identified by an in-house manufactured Excel-based macro. Lipid quantification was performed with MultiQuant, where peak areas of each identified lipid precursor ion were normalized to those of the corresponding internal standards.
